# Dissecting the Effects of Ischemia and Reperfusion on the Coronary Microcirculation in a Rat Model of Acute Myocardial Infarction

**DOI:** 10.1371/journal.pone.0157233

**Published:** 2016-07-08

**Authors:** Maurits R. Hollander, Guus A. de Waard, Lara S. F. Konijnenberg, Rosalie M. E. Meijer-van Putten, Charissa E. van den Brom, Nanne Paauw, Helga E. de Vries, Peter M. van de Ven, Jurjan Aman, Geerten P. Van Nieuw-Amerongen, Peter L. Hordijk, Hans W. M. Niessen, Anton J. G. Horrevoets, Niels Van Royen

**Affiliations:** 1 Department of Cardiology, VU Medical Center, Amsterdam, The Netherlands; 2 Institute for Cardiovascular Research, VU Medical Center, Amsterdam, The Netherlands; 3 Department of Anesthesiology, VU Medical Center, Amsterdam, The Netherlands; 4 Department of Molecular Cell Biology and Immunology, VU Medical Center, Amsterdam, The Netherlands; 5 Department of Epidemiology and Biostatistics, VU Medical Center, Amsterdam, The Netherlands; 6 Department of Physiology, VU Medical Center, Amsterdam, The Netherlands; 7 Department of Pathology and Cardiac Surgery, VU Medical Center, Amsterdam, The Netherlands; Emory University, UNITED STATES

## Abstract

**Background:**

Microvascular injury (MVI) after coronary ischemia-reperfusion is associated with high morbidity and mortality. Both ischemia and reperfusion are involved in MVI, but to what degree these phases contribute is unknown. Understanding the etiology is essential for the development of new potential therapies.

**Methods and Findings:**

Rats were divided into 3 groups receiving either 30 minutes ischemia, 90 minutes ischemia or 30 minutes ischemia followed by 60 minutes reperfusion. Subsequently hearts were ex-vivo perfused in a Langendorff-model. Fluorescence and electron microscopy was used for analysis of capillary density, vascular permeability and ultrastructure. Most MVI was observed after 30 minutes ischemia followed by 60 minutes reperfusion. In comparison to the 30’ and 90’ ischemia group, wall thickness decreased (207.0±74 vs 407.8±75 and 407.5±71, p = 0.02). Endothelial nuclei in the 30’-60’ group showed irreversible damage and decreased chromatin density variation (50.5±9.4, 35.4±7.1 and 23.7±3.8, p = 0.03). Cell junction density was lowest in the 30’-60’ group (0.15±0.02 vs 2.5±0.6 and 1.8±0.7, p<0.01). Microsphere extravasation was increased in both the 90’ ischemia and 30’-60’ group.

**Conclusions:**

Ischemia alone for 90 minutes induces mild morphological changes to the coronary microcirculation, with increased vascular permeability. Ischemia for 30 minutes, followed by 60 minutes of reperfusion, induces massive MVI. This shows the direct consequences of reperfusion on the coronary microcirculation. These data imply that a therapeutic window exists to protect the microcirculation directly upon coronary revascularization.

## Introduction

ST-segment elevated myocardial infarction (STEMI) is treated with direct revascularization by percutaneous coronary intervention (PCI). Although PCI has dramatically improved outcome in STEMI patients, it poses a new challenge. Despite a successful opening of the culprit vessel, in 40–50% of patients a part of the cardiac microvasculature remains non-perfused [1]. This is known as the no-reflow phenomenon and because it mostly affects the microvasculature, it is also referred to as microvascular injury (MVI)[2]. In the past decades it has become apparent that the presence of MVI is associated with a high morbidity and mortality [3, 4]. The worldwide prevalence of coronary artery disease affirms the urgency of adequate therapy. Unfortunately, despite several efforts no such therapy is available yet, because of limited knowledge on the pathophysiological mechanisms leading to MVI.

Evidently, the manifestation of MVI follows a period of ischemia and since MVI is predominantly located in the infarct core, it is theorized that ischemia plays a role in the development of MVI. This is demonstrated by Tarantini et al. who found a positive correlation of duration of ischemia and microvascular obstruction with magnetic resonance imaging (4). However the effects of ischemia on cardiomyocytes and endothelial cells are not equivalent. While infarct size mostly develops during ischemia[5, 6] the maximum extent of MVI is not found directly after ischemia, but rather develops over time after reperfusion. Several animal and clinical studies have shown that peak size of MVI lies >2 hours post infarction [7, 8]. This suggests that reperfusion has an additive harmful effect on the microvasculature. Because both phases are thought to play a role in the occurrence of MVI, the cascade is labeled as ischemia-reperfusion damage.

One of the hallmarks of MVI is the extravasation of erythrocytes and the development of intramyocardial hemorrhage (IMH)[9]. Experiments from over 4 decades ago show the presence of IMH in the area of no-reflow[10] and more recent studies have further linked CMR based MVI with the extravasation of erythrocytes[11, 12]. This contradicts the paradigm that MVI is based on obstruction (e.g. by erythrocyte plugging), and suggests that the microvascular integrity itself is affected (i.e. there is vascular leakage), already in the very early phases of reperfused STEMI. It is evident that both ischemia and reperfusion are essential for the occurrence of MVI, but it remains unclear to what degree both phases contribute to the damage of the microvascular wall and its integrity.

Previous pathophysiological studies on this topic have used several *in-vivo* models, either with ischemia alone or followed by reperfusion. Although this approach is insightful, it fundamentally cannot discriminate the effects of both phases. Ischemia-induced vascular leakage only becomes visible when flow (i.e. reperfusion) is administered, but *in-vivo* this induces possible additional damage. Also, only a few studies have focused specifically on vascular wall integrity and occurrence of IMH and most ultrastructural analyses are not qualitative.

This study is designed to compare the *in-vivo* effects of ischemia alone or ischemia-reperfusion in a rat model, with the addition of *ex-vivo* reperfusion and microsphere infusion in a Langendorff set-up. This allows the vascular leakage to become apparent, without introducing potential additional harmful effects of *in-vivo* reperfusion and it facilitates quantification of vascular leakage. Also, ultrastructural analysis of the microvasculature was performed via transmission electron microscopy (TEM).

## Methods

### Animals

All experiments were conducted with the approval of the Animal Welfare Committee of the VU University Amsterdam. 30 male Wistar rats (Harlan Laboratories, age 10 weeks, weight 300–400 gram) were acclimatized for two weeks and housed in groups of four animals. Group size was estimated based on 0.20 difference in mean, with an standard error of 0.15 and 0.8 power. Diet consisted of CHOW pallets and water *ad libitum*. Rats were housed in a temperature-controlled room (20–23°C; 40–60% humidity) under a 12/12h light/dark cycle starting at 6.00 am and inspected and weighed daily.

### Ischemia-reperfusion

Rats were divided in three groups. The first group (n = 10) received 30 minutes of cardiac ischemia and 0 minutes of *in-vivo* reperfusion, the second group (n = 10) received 90 minutes of cardiac ischemia and 0 minutes of *in-vivo* reperfusion, the third group (n = 10) received 30 minutes of cardiac ischemia, followed by 60 minutes of *in-vivo* reperfusion.

All rats were anesthetized with 5% isoflurane in an induction box and received a subcutaneous injection of buprenorphine (0.003mg/mL/100gBW). Subsequently rats were intubated and ventilated with 3% sevoflurane in oxygen enriched air (frequency 70/min). Rats were partly shaved and fixated on a heated table. Animals were connected to a 3 lead electrocardiographic continuous monitoring system (LabChart software, AdInstruments, Colorado Springs, CO). Chest wall was opened as described previously[13] and the pericardium was removed to bring the left anterior descending coronary artery (LAD) in sight. LAD was then ligated proximally, 1 mm below the left atrium, with a suture (5–0 Prolene) and a temporary ligation device. In the third group, after 30 minutes of ischemia, the ligation was removed, and animals received 60 minutes of *in-vivo* reperfusion.

### Langendorff model

The Langendorff set-up enables controlled *ex-vivo* coronary perfusion with a perfusion medium. The main part of the installation consists of a steel fixation cannula, a pressure transducer, a fluid access valve, a heat exchanger and a height adjustable heated flow-over reservoir. The reservoir was continuously filled by a peristaltic pump with perfusion medium. The perfusion medium was a modified Krebs-Henseleit buffer (MKHB), which consisted of (in mM) 118.5 NaCl, 4.7 KCl, 1.4 CaCl_2_, 25 NaHCO^3^, 1.2 MgCl_2_, 1.2 KH_2_PO_4_, and 11 glucose, which was freshly prepared and continuously oxygenated with carbogen gas (95% O_2_, 5% CO_2_), as described earlier[14]. Both the heat exchanger and the heated reservoir were set on such a temperature that the perfusion medium exiting the system was constantly 37°C. At the start of each experiment the system was cleaned, filled with fresh modified Krebs-Henseleit Buffer and air bubbles were filtered out. Also the pressure transducer was calibrated and monitored using Powerlab instrumentation (ADinstruments, Colorado Springs, CO).

After the above described periods of ischemia (and reperfusion) the aorta was cut distally of the aortic arch. In the 30–0 and 90–0 group, the temporary ligation device was then removed and the heart was excised as quickly as possible, taking care the myocardium and ascending aorta were not damaged. The excised heart was submerged directly in ice-cold perfusion medium to induce cardioplegia. Thereafter any unwanted tissue was removed and the ascending aorta dissected, leaving approximately 4mm for the fixation onto the cannula. The ascending aorta and the cannula were filled with perfusion fluid to prevent air embolisms. The heart was mounted onto the cannula and fixed with silk thread. The pressure of the perfusion medium was then slowly raised using a roller clamp until the heart started contracting again. Perfusion pressure was then steadily increased to 90 mmHg (Safedraw Transducer Blood Sampling Set, Argon Medical Devices, Texas, USA). Perfusion in the Langendorff setup was continued for 5 minutes.

### Fluorescent Microspheres

For the quantification of vascular leakage fluorescent microspheres (Fluospheres® carboxylate-modified, 0.1μm, 540/560; Life Technologies, Thermo Fisher Scientific, MA, USA) were used in 12 rats (4 per group). After mounting in the Langendorff setup, the hearts were perfused with MKHB until heart rate was stable. Subsequently a Fluosphere mix (3.6x10^12^ microspheres suspended in 1mL MKHB) was slowly added to the perfusion medium. After the addition of the mix, the heart was perfused with normal MKHB for 5 minutes, to remove any microspheres from inside the vasculature.

### Tissue preparation

After 5 minutes of Langendorff perfusion protocol, the heart was removed from the cannula and submerged in ice-cold MKHB to induce cardioplegia. Then the heart was transected into 5 slices of approximately equal thickness. Of each slice a biopsy (1x2mm) was taken from the center of the infarct zone, and one additional biopsy was taken from the posterior wall of the most basal slice, which served as a control. The biopsies were then fixed in 3% glutaraldehyde for ultra-structural analyses on the electron microscope. The remaining slices were snap frozen in liquid nitrogen for immunohistochemistry.

### Transmission Electron Microscopy

After adequate fixation in glutaraldehyde the biopsies underwent a secondary fixation with osmium tetroxide, dehydrated and embedded in epoxy resin (EPON, Miller-Stephenson, USA). After curing, 60–80 nm thick slices were cut with a Leica UC Ultra Microtome and placed on grids. Grids were then positively post stained with lead acetate and uranyl acetate. All samples were analyzed with a transmission electron microscope (CM 100 Bio, Philips, Eindhoven, The Netherlands), attached with a side-mounted TEM CCD camera (Morada G2, Olympus, Tokyo, Japan). Images were taken from capillaries at a magnification ≥5200x, with an exposure time of 200ms. For image processing and analysis iTEM software (OSIS, Tokyo, Japan) was used. Quantification of vessel characteristics was done in a blinded manner. Total vessel area, lumen area and nucleus area were manually delineated. Per animal 20 capillaries were analyzed. Endothelial cytoplasm area (including organelles) was calculated by subtracting lumen and nucleus area from the total vessel area. Mean cytoplasm thickness was calculated by dividing the cytoplasm area by the cytoplasm perimeter. Caveolae were counted and defined as total number per capillary. Chromatin density variation was determined by calculating the standard variation of grey values with the nuclear area.

### Immunohistochemistry

Frozen tissue samples were sectioned in slices of 5 micrometer. Sections of all samples were stained with hematoxilin and eosin (HE) and scanned with a Mirax slide scanner system using a 20× objective (3DHISTECH, Budapest, Hungary). Based on the HE images tissue samples with the largest area of infarction (containing infarct core) were selected for further immunohistological analysis. For the detection of smooth muscle cells, sections were stained using an antibody for smooth muscle actin (clone 1A4, Dako, 1:100). For the detection of endothelial cells (e.g. capillaries) an antibody for CD31 was used (PECAM-1, M20, Lot C0514, Santa Cruz Biotechnology, 1:50). Nuclei were stained with Hoechst (1:50000). Capillary density was calculated in a blinded manner as the average number of CD31^+^ SMA^-^ vessels per mm^2^, using a fixed frame of 200x200 mm^2^. Capillary density was calculated for the infarct core and the border zone, as well as controls.

### Statistics

To test for differences between three groups and control, a one-way analysis of variance (ANOVA) was used. When the ANOVA was significant, post-hoc analyses was performed according to Tukey’s method to compare between groups. Only significant post-hoc p-values are depicted in the figures. All results were considered statistically significant if the two-sided *P*-value was <0.05. For separate comparison of means between two groups a student’s t-test was used. Statistical analysis was performed with Statistical Package for Social Sciences software (SPSS 20.0 for Windows, SPSS Inc).

## Results

### Vascular leakage

**Fig 1:**
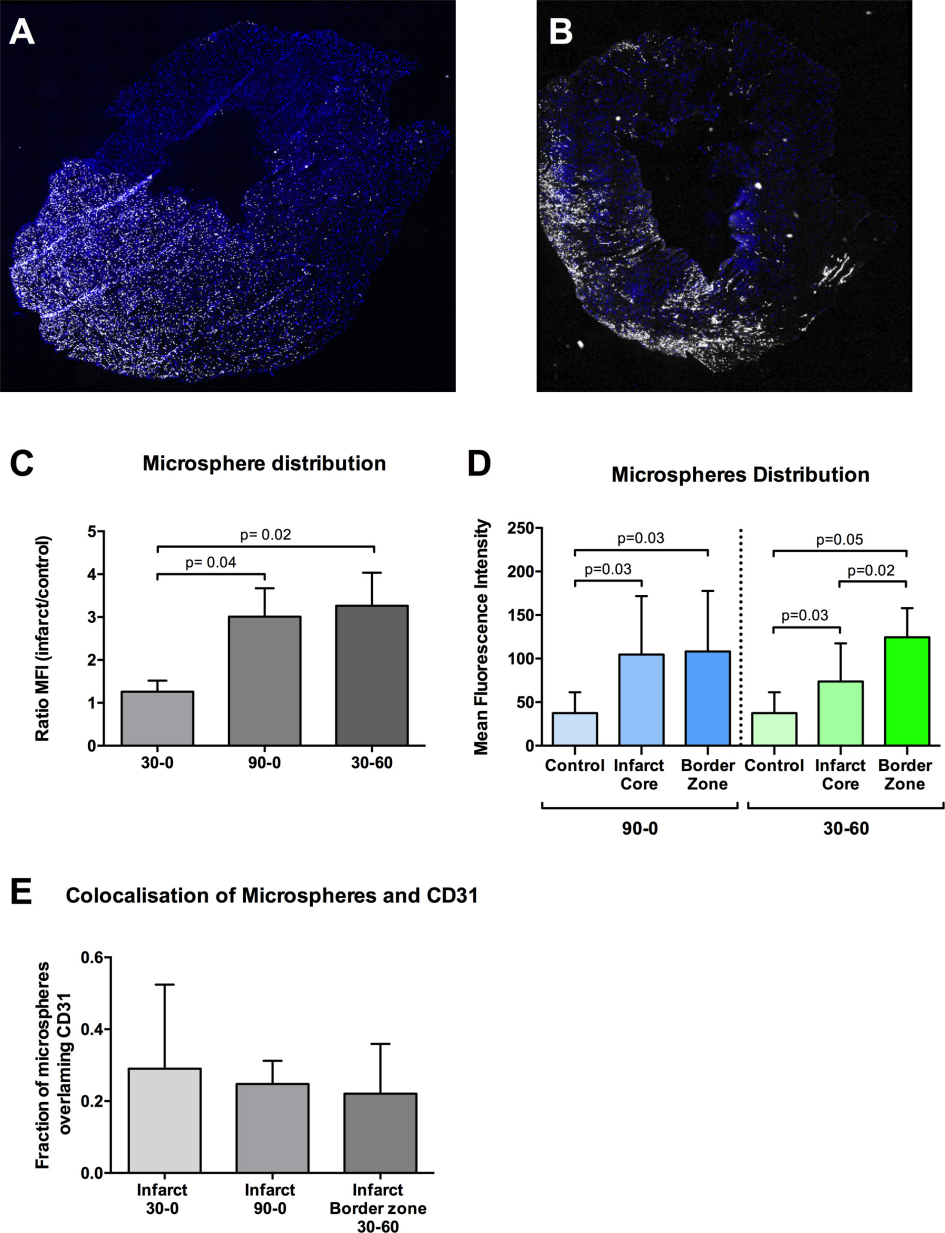
Distribution of fluorescent microspheres. **A-B** Images of transverse slices of the left ventricle, showing distribution of fluorescent microspheres (white). Nuclei are stained with Hoechst (blue). Red arrow: infarct zone, asterisk: control zone. **A-B** represents group 90–0 and 30–60 respectively. White bars represent 1mm. **C** Distribution of MFI within the 90–0 and 30–60 group **D** Mean fluorescent intensity (MFI) of fluorescent microspheres, expressed a ratio between infarct area and control area. **E** Pixel-based colocalisation analysis of fluorescent microspheres and CD31 positive tissue for extravasation.

Vascular leakage was quantified ex vivo by the detection of fluorescent microspheres extravasation in a Langendorff perfusion setting after excision of the infarcted rat hearts from the 3 groups. Mean fluorescent intensity (MFI) of the microspheres was calculated in the infarct zone and control zone (Fig 1A and 1B), and expressed as a ratio between both zones (Fig 1C) In the 30–0 group MFI was comparable with control (ratio 1.2). The 90–0 group showed a 3 fold increase of MFI in the infarct zone as compared to control. This increase was also present in the 30–60 group, where the ratio between infarct and control was comparable to the 90–0 (Fig 1C).

In the 30–60 group microspheres were found to be heterogeneously distributed in the infarct zone. Mean fluorescence intensity in the border zone of the infarct area in the 30-6- group was significantly increased, as compared with core of the infarct area (p = 0.02; Fig 1D). This difference was not found in the groups that underwent 30 or 90 minutes of ischemia, without *in-vivo* reperfusion.

All vascular lumina were devoid of microspheres, indicating successful coronary rinsing with normal perfusion fluid after perfusion with microsphere mix. Pixel-based colocalisation of microspheres and CD31 positive cells were similar in all groups (≈25%), indicating the extravasation of microspheres (Fig 1E).

### Capillary density

**Fig 2:**
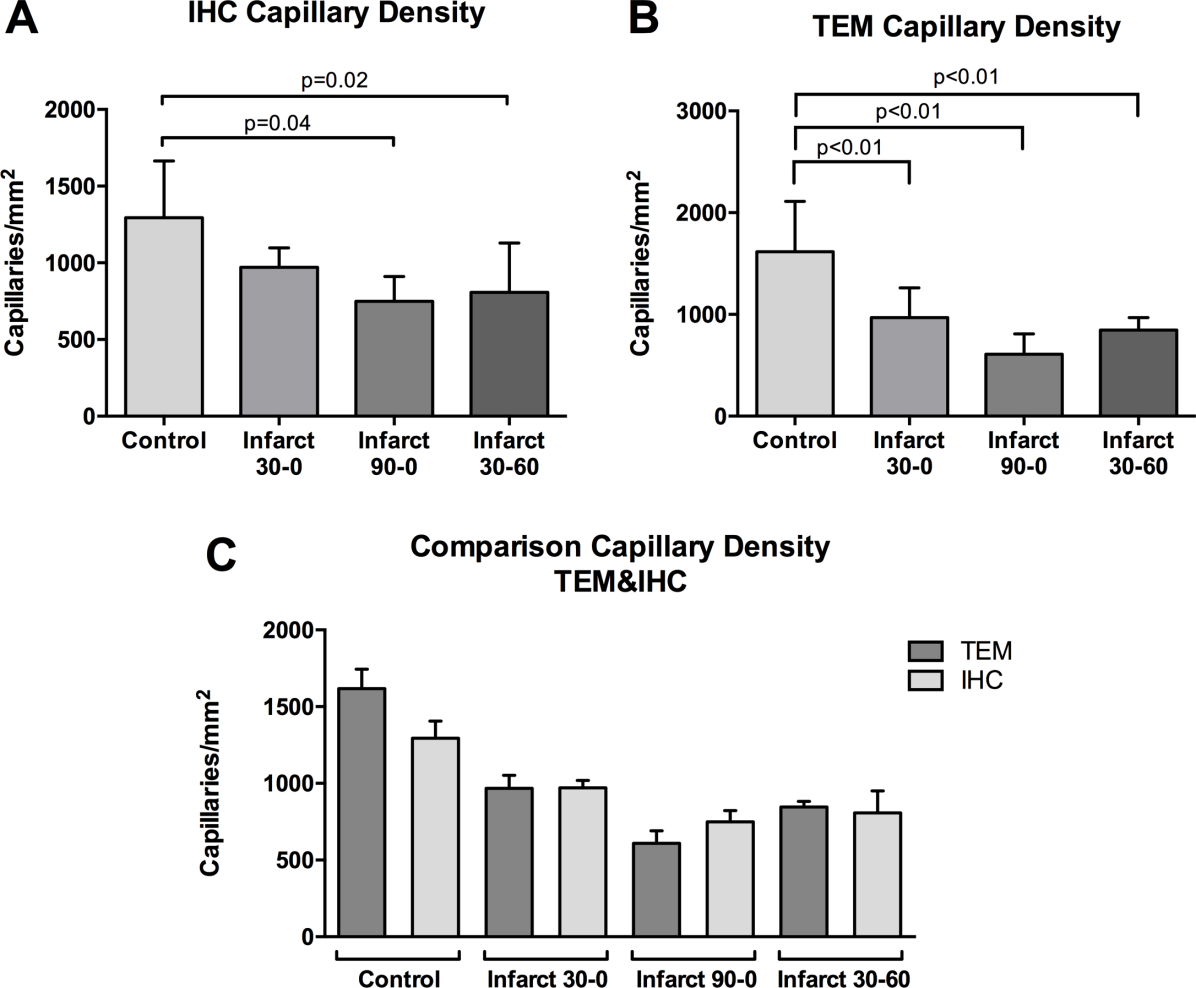
Capillary density. Immunohistochemical analysis of frozen tissue samples. **A** Quantification of capillary density by immunohistochemistry (IHC). **B** Quantification of capillary density by Transmission Electron Microscopy (TEM). **C** Comparison of capillary density between TEM and IHC. Capillary density is expressed as the number of capillaries per square millimeter.

Using immunohistochemistry, the mean number of CD31^+^ SMA^-^ capillaries (per square millimeter (i.e. capillary density) was quantified in both infarct area and control area in all groups. IHC based capillary density in control samples was equal in all groups (p = 0.36)(Fig 2A). Within groups capillary density was comparable in 30–0. In the 90–0 and 30–60 group there was a significant decrease in the capillary density in the infarct zone, as compared with control (1283±57.9 vs 638.1±66.3, p = 0.02).

Capillary density was also quantified using transmission electron microscopy and defined as the number of visible capillaries per square millimeter. The TEM based capillary density was comparable between control samples of all groups (p = 0.71). In the infarct tissue the capillary density differed between groups. In the 30–0 group the infarct zone capillary density was 1586±435, which was statistically comparable to the 90–0 group (1716±283) and 30–60 group (1185±172.6). Only the 30–60 group showed a significant decrease in capillary density compared to control (p<0.01, Fig 2B).

Interestingly, in all groups capillary density as was lower when measured with IHC, as compared to TEM (p<0.01; Fig 2C).

### Ultrastructural analysis

**Fig 3:**
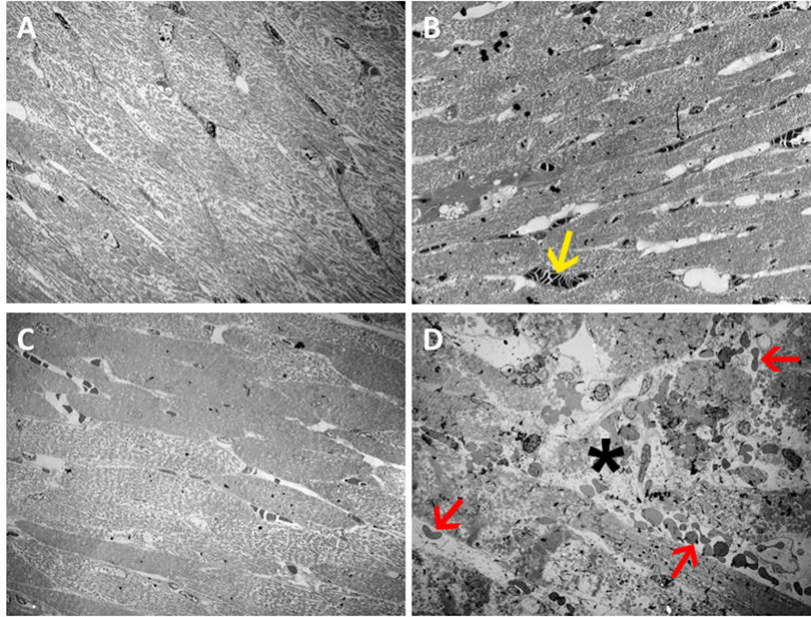
Overview of TEM Images. Transmission electron microscopy images, overview images, magnification 560x. **A** Images from control samples. **B-D** Images from infarct zone samples. **B** 30–0 group, **C** 90–0 group, **D** 30–60 group. Massive tissue damage is visible (asterisk), with extravasation of erythrocytes (red arrow), and intraluminal erythrocytes (yellow arrow). White bar represents 10 micrometer.

**Fig 4:**
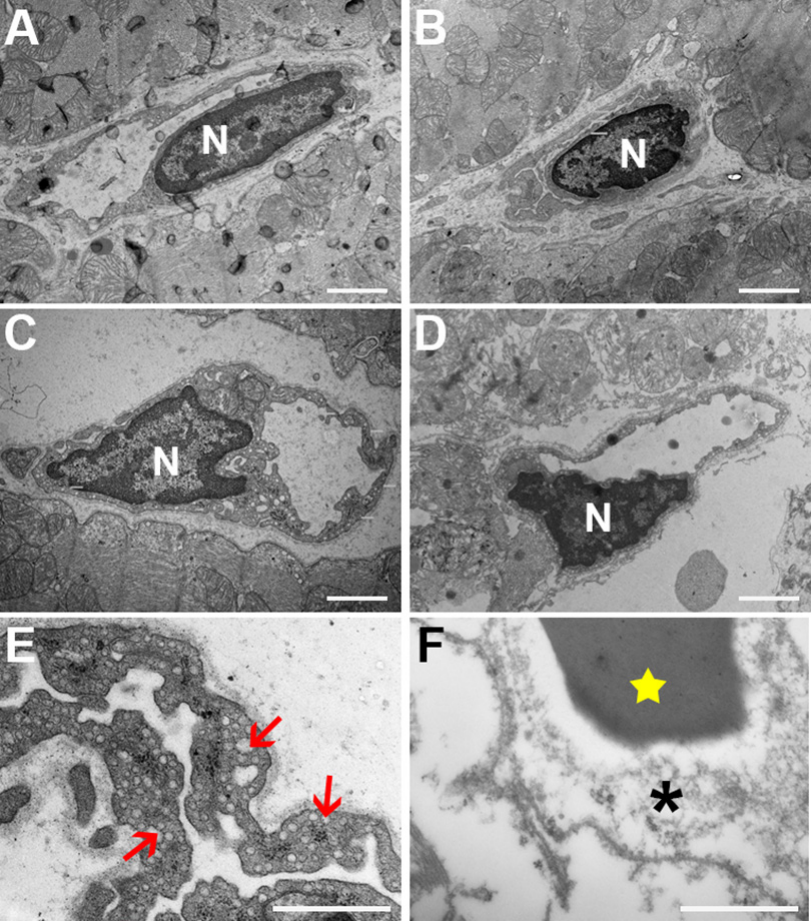
Ultrastructural images. Magnification A-D 10500x. **A** Capillary in the control tissue. **B** Capillary from infarct zone of the 30–0 group. In both A and B an intact continuous vessel wall can be seen. **C** Image depicts the 90–0 group and shows activated endothelium with abundant presence of caveolae and vesicles. Cytoplasm shows great variety in thickness, with multiple protrusions (white triangles). **D** Capillary from group 30–60 which shows severely damaged vessel wall with disruption of the basal membrane. **E** and **F** show detailed images with numerous caveolae (red arrows) and destruction of the vessel wall (asterisk). Also an erythrocyte can be seen (star). Nucleus indicated with the letter N. White bars represent 2 micrometer. Magnification E-F 24.500x.

Transmission electron microscopy images showed no clear pathologic characteristics of vascular structures in the 30–0 group, which received 30 min ischemia without reperfusion (Fig 3A). All blood vessels were intact and showed complete absence of blood cells, which affirms adequate and complete Langendorff perfusion. Cardiomyocytes were normally aligned and showed clear Z- and M-bands. Mitochondria also showed normal structure and size. Overall there were no differences with the paired control samples. In the infarct zone of the 30–0 group a similar pattern could be seen, with erythrocytes occasionally visible, but always within the vasculature (Fig 3B). In the 90–0 group which received 90 minutes ischemia without reperfusion, blood vessel integrity was also intact. Furthermore, lumina were completely empty, indicating successful Langendorff perfusion with transparent buffer. Macroscopically, there were no signs of ischemic damage to cardiomyocytes at 90 minutes of ischemia (Fig 3C). In the 30–60 group cardiac tissue in the infarct zone was clearly disrupted, showing extensive damage with misalignment of cardiomyocytes, disruption of capillaries and massive extravasation of erythrocytes (i.e. intramyocardial hemorrhage)(Fig 3D). When studied in more detail, endothelial cells, displayed normal morphology in the control samples and in the infarct zone of the 30–0 group (Fig 4A and 4B). In the infarct zone of the 90–0 group capillary endothelial cells showed clear cytoplasmatic blebbing and an increase in number of caveolae, both signs of endothelial cell activation (Fig 4C and 4E). Besides this, nuclear characteristics also showed marked alteration. In the 30–60 group, clear morphologic alterations were observed. Mitochondria were swollen and showed loss of cristae. Furthermore, mitochondria contained electron dense depositions as a sign of irreversible damage (Fig 4D). The nuclei of endothelial cells also showed signs of cell death, namely chromatin condensation. Vessel walls were generally thinner (Fig 4F) than normal and in some areas showed severe damage and loss of vascular integrity with erythrocytes located in the perivascular space (i.e. intramyocardial hemorrhage). No extravascular lymphocytes were observed. Ultra-high magnification TEM imaging allowed detailed quantitative analysis of capillaries, of which results are listed below.

### Mean vessel and lumen area

**Fig 5:**
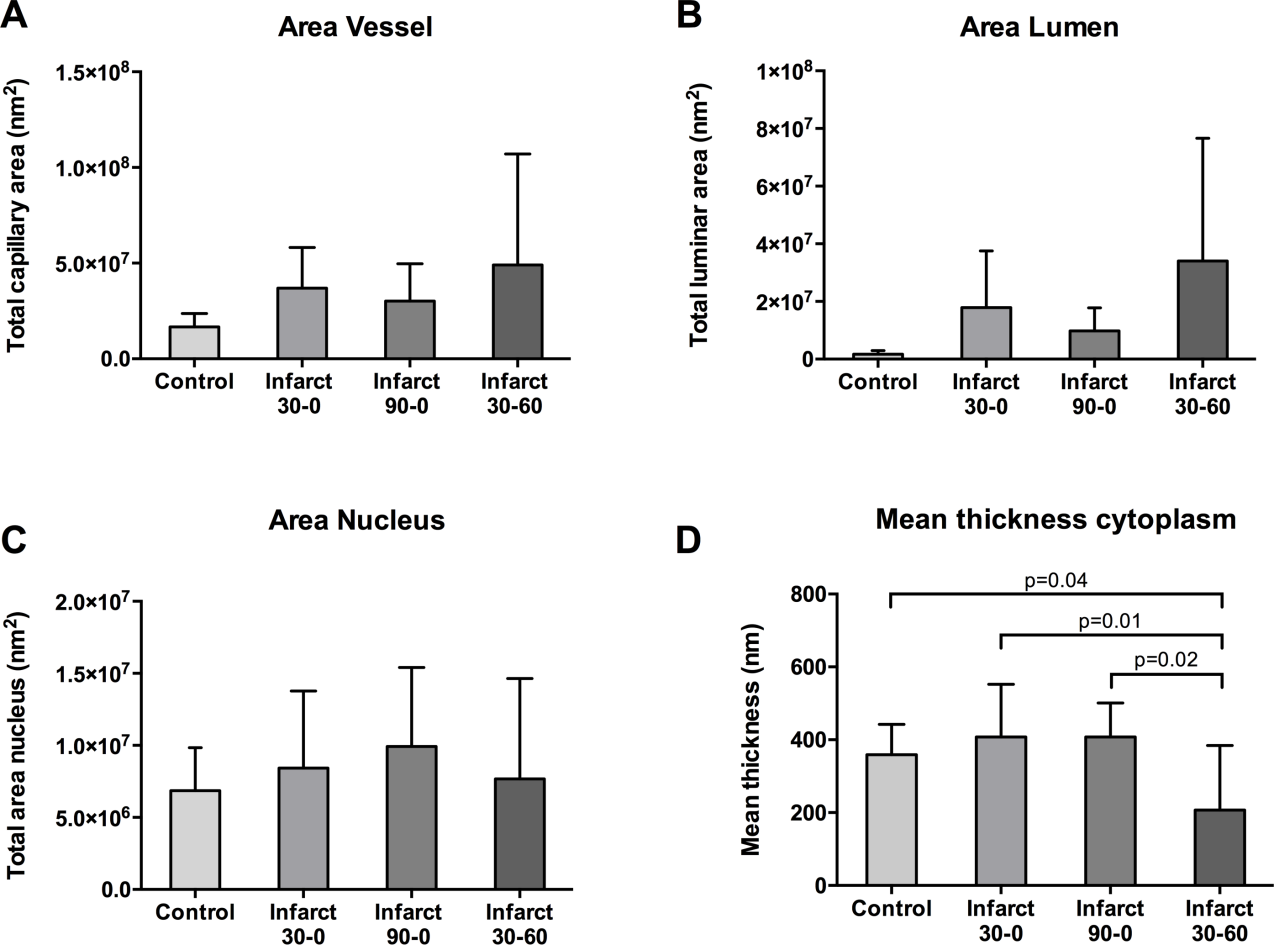
Area measurements of microcirculation. **A** Total vessel area in nm^2^ (including cytoplasm, lumen and nucleus). **B** Area of vessel lumen in nm^2^. **C** Area of endothelial nuclei in nm^2^. **D** Mean thickness of the capillary cytoplasm in nm.

When different groups were compared no significant difference in mean vessel area in the infarct zone and the control zone was observed (infarct: 36.8±8.6 nm^2^ vs 30.2±7.9 nm^2^ vs 49±20 nm^2^; control 19.56±3.0 nm^2^ vs 13.7±3.1 nm^2^ vs 19.7±4.0 nm^2^, p = 0.33; Fig 5A). When all groups were taken together, mean infarct vessel area was significantly higher than mean control vessel area (P = 0.04). This significance was not reached when infarct and control were compared within groups (Fig 5A). Between groups, lumen area showed no difference (17.9±8.3 nm^2^ vs 9.75±3.4 nm^2^ vs 35.6±15.2 nm^2^, NS; Fig 5B). In all groups the infarct zone lumen area was significantly increased as compared to the control zone (mean difference 15.7 nm^2^, 8.8 nm^2^ and 33.8 nm^2^, p = 0.012, p = 0.046 and p = 0.011 respectively; Fig 5B).

### Endothelial cytoplasm and nucleus area

Between groups there was no difference in endothelial cytoplasm area in the infarct zone (infarct: 13.4±3.3 nm^2^, 9.0±2.6 nm^2^ and 7.8±3.3 nm^2^, NS; data not shown). Also within groups there was no difference in total cytoplasm area between infarct zone and control (mean difference 4.4 nm^2^, 2.0 nm^2^ and -3.7 nm^2^, NS; data not shown). Compared between groups there were no differences in the nucleus size in the infarct zone (infarct 8.4±2.6 nm^2^, 9.9±2.2 nm^2^ and 7.7±2.8 nm^2^, NS; Fig 5C). Also within groups there was no difference in nuclear size between infarct zone and control (mean difference 0 nm^2^, 4.2 nm^2^ and 1.2 nm^2^, all NS; Fig 5C).

When corrected for vessel circumference there was no difference in mean cytoplasm thickness (i.e. vessel wall thinkness) between groups in the control zone (Fig 5D). The mean cytoplasm thickness in the infarct zones of the 30–0 and 90–0 group was also comparable. In the 30–60 group however mean cytoplasm thickness is significantly lower than the other groups (Fig 5D).

### Endothelial cell nuclei characteristics

**Fig 6:**
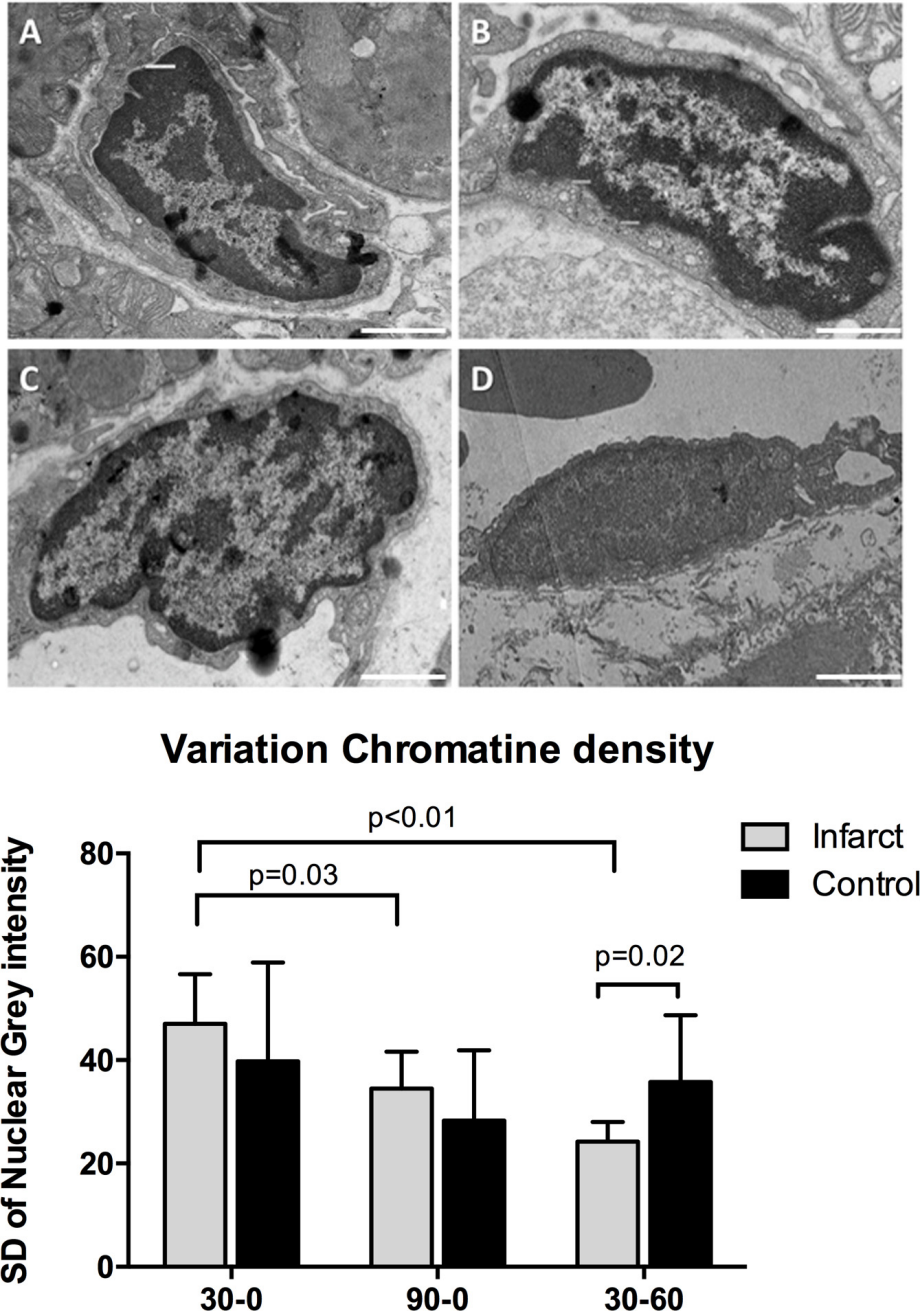
Endothelial cell nuclei characteristics. Top four images exemplify typical TEM images of infarct zone samples from different groups. **A-D** represent the control, and infarct zones of the 30–0, 90–0 and 30–60 group respectively. Magnification 12500x White bars represents 2 micrometer. **E** Quantification of chromatin density variation, using iTEM software.

Although nuclei showed no differences in average size, their appearance on TEM imaging was different between groups. Nuclei in the 30–0 group had a normal morphology and were similar to control samples (Fig 6A and 6B). In the 90–0 group endothelial nuclei showed some signs of early apoptosis, with DNA condensation on nucleus borders (Fig 6C). In the 30–60 group the morphologic changes were most dramatic, with a more homogenous appearance, indicative for end-stage apoptosis (Fig 6D). Control samples showed no signs of DNA degradation. DNA degradation resulted in less diverse chromatin distribution, which resulted in an uneven spread of grey intensity within the area of the nucleus between groups. The 30–0 group had a significantly higher chromatin density variation (CDV) than the 90–0 group (30–0: 50.5±9.4, 90–0: 35.4±7.1, p = 0.03). The 30–60 group had the lowest CDV (23.7±3.8), which was significantly lower than both the 30–0 group (p≤0.01) and control (p = 0.02), but not compared to the 90–0 group (p = 0.08) (Fig 6E).

### Cell-cell junctions and caveolae

**Fig 7:**
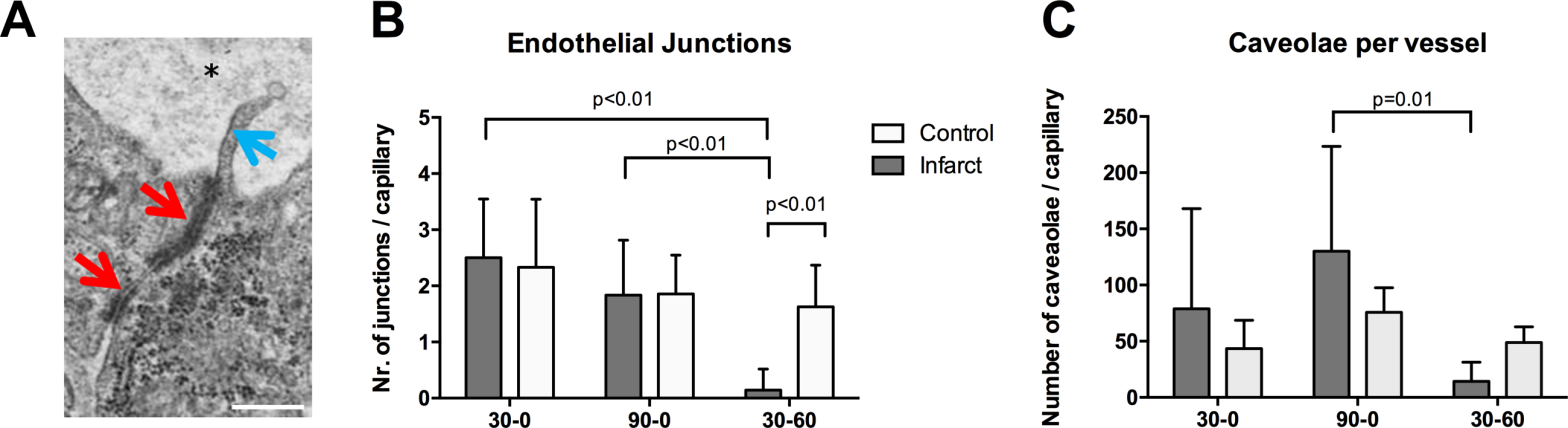
Endothelial junctions and caveolae. Quantification of endothelial cell-cell junctions. **A** Typical TEM image of a part of two endothelial cells with cell-cell junctions (red arrows). Protruding into the lumen of the capillary (asterisk) marginal fold can be seen (blue arrow). White bar represents 100 nanometer. Magnification 17500x. **B** Quantitative analysis showing the mean number of cell-cell junctions per capillary in the infarct zones in all three groups. **C** Mean number of endothelial caveolae per capillary, as determined with transmission electron microscopy.

Vascular integrity of the microcirculation is largely dependent on the endothelial cell-cell junctions, which limit interendothelial leakage of plasma components to the subendothelial space. At the capillary level these junctions can easily be seen with transmission electron microscopy, and quantified (Fig 7A). Number of visible junctions per capillary varied significantly between groups. The infarct zone of the 30–0 group showed a mean number of junctions per capillary of 2.5±0.6, which was comparable with the 90–0 group (1.8±0.7, NS; Fig 7B). In contrast, the 30–60 group showed the lowest number of junctions per capillary (0.15±0.2), which was significantly lower than both the 90–0 group (P<0.01) and the 30–0 group (P<0.001), indicating lower vascular integrity (Fig 7B). Transendothelial transport is facilitated by caveolae (transcytosis). The endothelial cells in the infarct area of the 30–60 group had significantly less caveolae than the 90–0 group (p = 0.01, Fig 7C). In the 90–0 there appeared an increase in number of caveolae which did not reach significance due to high variability. The average size of the caveolae was comparable between groups (30–0: 100.2±12.35 nm, 90–0: 97.14±7.8 nm, 30–60: 81.25±26 nm, NS; Fig 7C).

## Discussion

In this study, we aimed to investigate the specific effects of ischemia and reperfusion on the coronary microcirculation in an experimental rat model. By using the combination of *in-vivo* ischemia (and reperfusion) and controlled ex-vivo perfusion in a Langendorff set-up, we could discriminate between the effects of these two phases. We show that compared to ischemia alone, the addition of reperfusion inflicts major additional damage on endothelial cells and increases vascular permeability. In the *in-vivo* reperfusion group the capillary wall thinned significantly, with a loss of visible cell-cell junctions.

Ischemia-reperfusion damage has been well described in literature and its impact on infarct size and left ventricular remodeling is paramount [15, 16]. The effects on the microcirculation however are less well described. Recently we observed in a porcine ischemia-reperfusion model major loss of the vasculature, accompanied by intramyocardial hemorrhage (IMH)[11]. However, the etiology of this vascular leakage is still not clear. In literature it is postulated that the post-ischemic microvasculature–which relies on anaerobic metabolism- cannot cope with sudden influx of oxygen, which is induced by reperfusion[17]. As a consequence reactive oxygen species are formed, which are believed to induce cell death. However, in a clinical setting ischemic post-conditioning showed no beneficial effect[18], which suggests other mechanisms are triggered after ischemia is relieved. Other factors that are suggested to play a role in ischemia-reperfusion damage are the influx of leukocytes, plasma proteins and the activation of the complement.

When the effects of ischemia-reperfusion on vascular integrity are studied *in-vivo*, it is however impossible to clearly distinguish the specific effects of ischemia and reperfusion. Without reperfusion vascular leakage does not become visible. This was recently elegantly shown by Fernández-Jiménez et al, who studied edema development during ischemia-reperfusion in pigs[19]. Also, intramyocardial hemorrhage does not occur in the infarct core (or only to a very limited extent) if the epicardial vessel is not reopened. However, the absence of vascular leakage or hemorrhage does not exclude ischemic damage to the vasculature. The Langendorff set-up allowed us to detect ischemia induced vascular permeability, without introducing potential additional harmful effects of *in-vivo* reperfusion (caused by thrombocytes, leukocyte invasion[20] or erythrocyte extravasation[12]). Data from our study show that *in-vivo* reperfusion has a clear and large additional damaging effect on the coronary microcirculation. The increased vascular permeability in the 30–60 group is in line with other studies that report peak microvascular obstruction after >60 minutes of reperfusion [7, 8].

### Endothelial cells

In the acute setting, ischemia leads to morphologic changes of the endothelium. In all groups the lumina of the infarct-related capillaries were enlarged. This contradicts some studies that report vasoconstriction [21–23], but these studies are based on pressure measurements or done in hindlimb models. The enlarged lumina in our study maybe the result of transient post-occlusive hyperemia[24]. Ambrosio et al. showed that after coronary ischemia, regional microvascular blood flow increased during the early phase of reperfusion and remained upregulated even after 210 minutes of reperfusion[25]. This study also showed with transmission electron microscopy that after reperfusion the cytoplasm of endothelial cells is thinned and disrupted with a noticeable absence of transcytotic vesicles i.e. caveolae. Our data shows identical findings in the 30–60 group, underlining the additional destructive effects of reperfusion on the microvasculature.

### Chromatin condensation

We find considerable alterations in nuclear chromatin distribution of endothelial cells, most pronounced in the 30–60 group, followed by the 90–0 group and normal in the 30–0 group. Chromatin condensation (i.e. clumping) is indicative for irreversible cell damage and characterized by an irregular distributed chromatin in the nucleus. These changes are well described[26], but remain mostly qualitative. Some scoring techniques for electron microscopy images have been proposed[27, 28]but these remain subjective to some degree. There are other, more quantitative methods[29] but these rely on complex fluorescence imaging. We present a simple but quantitative method to investigate chromatin condensation and nuclear distribution, by analyzing the dispersion of grey values with the nucleus.

To some degree, the endothelial cells in the 90–0 group showed also vascular and endothelial damage, but not to the extent of the 30–60 group. The 90–0 group did show an increase in the number of cytoplasmatic caveolae, which implies endothelial activation. The exact role of these vesicles remains unclear, but they are associated with cardiac pathology and currently widely studied[30, 31]. Alteration in the number of caveolae after ischemia was already described by Kloner several decades ago[10]. More recently it is suggested that the formation of chains of caveolae creates transcytoplasmic pores, that accelerate vascular permeability[32] and that caveolae are also an essential component of endothelial cell signaling after abrupt reduction of flow[33].

### Vascular leakage

In our study vascular leakage was quantified using the ex-vivo administration of fluorescent microspheres. Most of the microspheres (0.1 micrometer) extravasated in the 90–0 and 30–60 group. Interestingly, in the 30–60 group the infarct core contained hardly any fluorescent spheres and the border zone showed to have a 3-fold increase in MFI, as compared to control. This heterogeneous distribution was not seen in the other groups. This means that after 90 minutes of ischemia alone, the spheres were able to reach the complete infarct area, but after 30 min of ischemia followed by 60 minutes of reperfusion, the infarct core was not accessible for the spheres, indicating no-reflow. This very much resembles the clinical situation, showing increased magnetic resonance imaging contrast accumulation in the border zone of the infarct and absence on contrast in the core of the infarct[11]. The increased vascular permeability was accompanied by a decrease in the number of visible cell junctions per vessel after 60 minutes *in-vivo* reperfusion. These junctions play an important role in vascular integrity[34]. Comparable data on junction loss is described in an ischemia-reperfusion model in the lung[35], brain[36] [37]and kidney[38]. Interestingly the microspheres extravasation in the 90–0 group was not accompanied with a loss of visible junctions. Possibly the very small size of the microspheres (100nm) allowed for early extravasation, even when number of endothelial cell-cell junctions is not visibly decreased. Alternatively, they can be transported via transcytosis based on the apparent increased number of caveolae specifically in the 90–0 group.

### Capillary density

Functional capillary density is known to decrease as early as 20 min after coronary artery clamping followed by short reperfusion [39]. In our study we don’t see a decrease of capillary density after 30 minutes of ischemia. Capillary density was found to be significantly decreased in the infarct zone of the 30–60 group, compared to control and measured by both immunohistochemistry and TEM. In the 90–0 group, a similar effect was also seen with IHC indicating ischemia alone does affect the microvasculature. Interestingly, in our study, capillary density did differ between imaging techniques. In all groups the capillary density measured by TEM was significantly higher than measured by IHC (Fig 2C), which could be explained by a change in CD31 activity/availability or an incomplete antibody binding with CD31 molecules and/or an partial binding of secondary antibodies.

### Clinical implications

Although first established in preclinical setting, MVI is nowadays acknowledged as a serious complication of coronary revascularization in patients with acute myocardial infarction. MVI has a poor prognostic value and is associated with adverse cardiac remodeling and mortality[40]. Several therapies has been investigated in clinical setting, which rely on different pathophysiological processes, such as limiting oxidative stress[41, 42], apoptosis suppression[43], and reduction of embolization by thrombotic material[44–46]. Although some promising results have been obtained, none of these therapies are currently standard practice, possibly based on the questionable additive value of MVI therapy. Data from this study suggests that at the moment of revascularization, the cardiac microvasculature is still mostly intact. This implies that vascular protecting strategies could be beneficial on top of mechanical revascularization. Recent findings linking MVI to intramyocardial hemorrhage[47] suggests protecting vascular permeability could be a potential therapy. Endothelial integrity and vascular permeability have been studied in a wide variety of research fields, such a pulmonary edema, oncology and the blood-brain barrier[48–50]. This has produced multiple candidates for the therapy of microvascular injury, such as the use of imatinib [51]statins [52] and complement inhibitors[53], Furthermore, the presence of intramyocardial hemorrhage[11] suggests the current use of peri-procedural anticoagulant drugs in patients undergoing angioplasty needs to be further investigated.

There are some limitations to the current study. First, microsphere size was very small (0.1 μm), which is considerably smaller than erythrocytes (±7μm). Microsphere extravasation may therefore not be extrapolated to intramyocardial hemorrhage in animals that did not have *in-vivo* reperfusion. Because vascular leakage increases gradually microsphere extravasation can therefore be seen as early loss of endothelial integrity. In a study assessing reperfusion damage in mice, it is shown that infarct size is finalized after 60 minutes, as compared to 24 hours [54]. Endothelial apoptosis also follows this time course, with a peak at 1 hour after ischemia[55]. This does not mean however that vascular integrity is stable 60 minutes after reperfusion. Our data shows that 60 minutes of reperfusion have an additional harmful effect on endothelial cells, as compared to ischemia alone. However, there are several studies indicating that the extent of MVI reaches it maximum in the period after 1h of reperfusion [7, 8].

## Conclusion

In the acute setting of myocardial infarction reperfusion is mostly responsible for the damage to the coronary microcirculation, inducing endothelial cell dysfunction and death, and increased vascular leakage, whereas ischemia alone has limited effect. This implies that a therapeutic window is available to maintain endothelial integrity directly upon coronary revascularization.

## Abbreviations

CD31: Cluster of differentiation 31
CMR: Cardiac Magnetic Resonance
HE: Hematoxilin & Eosin
IHC: Immunohistochemistry
IMH: Intramyocardial Hemorrhage
LAD: Left Anterior Descending artery
MFI: Mean fluorescence intensity
MKHB: Modified Krebs-Henseleit buffer
MVI: Microvascular Injury
PCI: Percutaneous Coronary Intervention
PECAM-1: Platelet endothelial cell adhesion molecule 1
STEMI: ST-segment Elevated Myocardial Infarction
TEM: Transmission Electron Microscopy
