# Correction: Dissecting the Effects of Ischemia and Reperfusion on the Coronary Microcirculation in a Rat Model of Acute Myocardial Infarction

**DOI:** 10.1371/journal.pone.0166809

**Published:** 2016-11-15

**Authors:** Maurits R. Hollander, Guus A. de Waard, Lara S. F. Konijnenberg, Rosalie M. E. Meijer-van Putten, Charissa E. van den Brom, Nanne Paauw, Helga E. de Vries, Peter M. van de Ven, Jurjan Aman, Geerten P. Van Nieuw Amerongen, Peter L. Hordijk, Hans W. M. Niessen, Anton J. G. Horrevoets, Niels Van Royen

The tenth author’s name is spelled incorrectly. The correct name is: Geerten P. van Nieuw Amerongen.

## References

[1] Hollander MR, de Waard GA, Konijnenberg LSF, Meijer-van Putten RME, van den Brom CE, Paauw N, et al (2016) Dissecting the Effects of Ischemia and Reperfusion on the Coronary Microcirculation in a Rat Model of Acute Myocardial Infarction. PLoS ONE 11(7): e0157233 doi: 10.1371/journal.pone.0157233 27391645 10.1371/journal.pone.0157233 PMC4938574

